# Extracorporeal tumor cell filtration during extended liver surgery: first clinical use of leukocyte depletion filters – a case series

**DOI:** 10.1186/1477-7819-11-159

**Published:** 2013-07-17

**Authors:** Karl J Oldhafer, Gregor A Stavrou, Marcello Donati, Philipp Kaudel, Nils R Frühauf

**Affiliations:** 1 Department of General and Abdominal Surgery, Asklepios Hospital Barmbek, Rübenkamp 220, 22293 Hamburg, Germany; 2 Department of General and Abdominal Surgery, Johannes-Gutenberg University, Langenbeckstraße 1, 55131 Mainz, Germany; 3 Ärztekammer Niedersachsen, Berliner Allee 20, 30175 Hannover, Germany

**Keywords:** *Ex situ* resection, Leukocyte depletion filter, Liver metastasis, Oncologic surgery, Veno-venous bypass

## Abstract

**Background:**

During oncologic surgery, intraoperative manipulation of tumor tissue is almost unpreventable and causes a high risk of tumor cell dissemination into venous blood. A tumor cell-reducing effect of leukocyte adhesion filter systems has been shown under *in vitro* conditions.

**Methods:**

In a first clinical attempt, leukocyte adhesion filters were integrated into veno-venous bypass systems in four patients undergoing extended liver surgery for secondary hepatic malignancies.

Practicability, handling, and safety aspects as well as potency of cell removal and clinical side effects of the filter system were analyzed.

**Results:**

All patients tolerated the application of the system without problems during operative and postoperative follow-up. Immunohistochemical staining of perioperative blood samples detected cytokeratin positive (CK+) cells in three cases during the hepatic mobilization.

**Conclusions:**

Effectiveness of CK+ cell depletion and safety of the procedure was shown. The presented surgical technique represents a safe and innovative tool; however, clinical significance has to be examined in a larger patient cohort.

## Background

In many studies the prognostic relevance of circulating tumor cells in patients undergoing resection of primary and metastatic colorectal cancer has been demonstrated [1,2]. In addition to preoperatively existing circulating tumor cells, manipulation of tumor tissue during oncologic surgery may result in an extra tumor cell release into the systemic blood circulation. Therefore, intraoperative surgical manipulation of tumors could influence the prognosis of the underlying malignant diseases [3-5]. A simple method to prevent such perioperative cell dissemination in patients with primary colorectal cancer was described in the 1960’s, the so-called ‘no-touch’ surgery, with early vascular closure before preparation and resection [6]. In patients undergoing liver resection for primary or secondary malignancies, prevention of intraoperative tumor cell dissemination is difficult to obtain. The high incidence of mechanically-induced intraoperative tumor cell dissemination might be caused by the anatomy and the need of extensive mobilization before possible occlusion of the venous drainage [7]. Therefore, surgical attempts such as the ‘anterior approach’ were developed [8,9]. As surgical mobilization is unpreventable in most attempts, additional techniques to reduce the release of tumor cells into the systemic circulation should be considered. We have previously shown that cell filtration of blood drained from veins located close to the tumor could be a useful option [10]. As shown in previous studies, special filter mediums used for leukocyte depletion are able to remove tumor cells under *in vitro* conditions [11]. Comparable filter systems are used in cardiac surgery combined with a heart-lung machine and in blood banks for preparation of stored blood units [12].

We present a clinical pilot study in four patients suffering from secondary hepatic malignancies. Patients underwent extended liver surgery with leukocyte depletion filters integrated within the extra corporal veno-venous bypass system.

## Methods

After preclinical testing in a porcine model [13] and approval of the local ethics committee (Hannover Medical School) for first clinical investigations, leukocyte adhesion filters (Leukogard LG-6, Pall^©^, Germany) were integrated into the bypass circuit (bio pump system BP80, BioMedicus^©^, Germany) in four patients undergoing extended liver surgery [14] due to colorectal metastasis with the need of extracorporeal veno-venous circulation (Table 1).

**Table 1:** Patient’s characteristics

**Case**	**Age/sex**	**Primary Tumor**		**Prior treatment before admission**		
		**Location**	**Stage**	**Surgery**	**CTX**	**RTX**
			**(UICC, TNM)**			
**1**	72/m	Colon	III	Hemicolectomy left	**+**	**-**
			T3N1M0			
**2**	66/f	Rectum	IV	Anterior rectum resection	**+**	**+**
			T3N0M1			
**3**	49/f	Colon	IV	Rectosigmoidectomy	**+**	**-**
			T3N2M1	Hemihepatectomy right + S III		
				Metastasectomy S II		
**4**	62/m	Rectum	IV	Anterior rectum resection	**+**	**-**
			T3N2M1	Segmentectomy S VII		

Patient data: localization, UICC, and TMN stage of primary tumor; prior treatment before admission: surgery, chemotherapy (CTX), and radiotherapy (RTX).

Potency of cell removal, clinical side effects and technical handling of the filter system were analyzed. Patient follow-up was performed up to 26 months after operation.

### Veno-venous bypass

**Figure 1:**
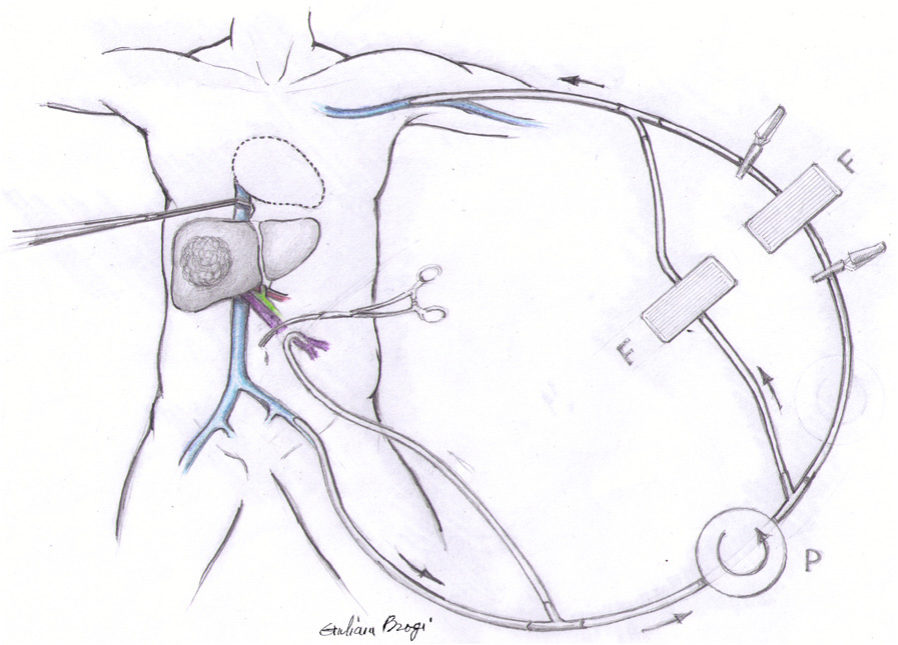
**Schematic drawing of the extracorporeal veno-venous bypass circulation system.** Portal, left femoral, and left subclavian veins are cannulated by flow catheters. A centrifugal bio pump system (BP80, BioMedicus^©^, Germany) is used **(P)**. Filter cartouches **(F)** are integrated in parallel.

The extracorporeal bypass system was implemented by puncture of the left subclavian and left femoral vein using high-flow catheters (14 F, Medos^©^, Germany) in the Seldinger technique. After laparotomy and dissection of the hepatoduodenal ligament the portal vein was additionally cannulated [15]. Leukocyte filters were integrated into the bypass circuit. The tubing system was pre-coated with 10% albumin-solution. After system setup, the extracorporeal circulation was started following simultaneous clamping of the inferior cava vein beneath the diaphragm (Figure 1); the flow rate was kept between 0.9 and 2.5 L/min. During bypass circulation all patients received heparin within an activated clotting time target of >150 s. Each 30 minutes of filtration the filter cartridges were replaced. Once hepatic mobilization finished, leukocyte depletion filters were removed for the remaining bypass time (individually depending on the surgical procedure).

### Filtration system

LG-6 (Leukogard 6®, Pall corp., NY, USA) is a leukocyte-depletion arterial blood filter, a latex-free device, with a polycarbonate surface. The maximum blood flow filtration rate is 6 L/min. It provides a three-stage system for gas separation with automatic venting system and has a 40 μm-pore polyester filter, disposable as a cartridge. The priming volume for this filter is 220 mL, with 3/8-inch barbed inlet and outlet ports.

### Surgical procedure

#### *Case 1*

**Figure 2:**
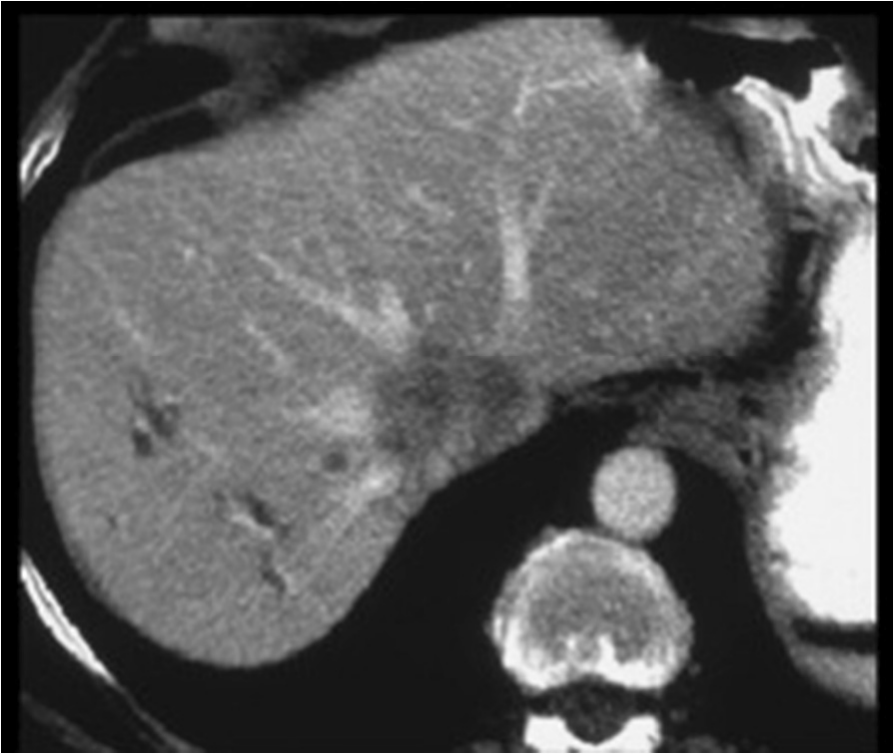
**CT scan showing the liver metastasis infiltrating the inferior vena cava and all three liver veins.** The tumor was mainly located in segment I.

**Figure 3:**
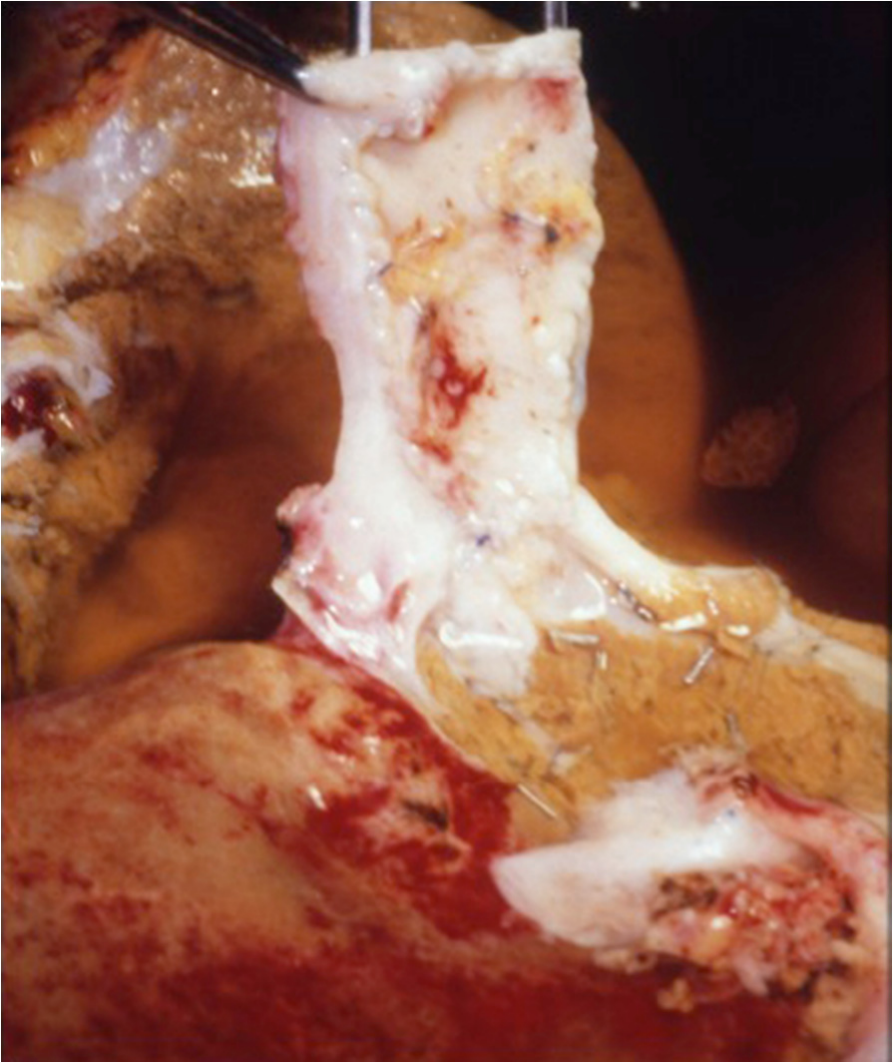
**A bench resection of segments I, IV to VIII was performed under *ex situ *conditions.** The inferior vena cava and the liver veins were reconstructed using parts of the more distal inferior vena cava which itself was replaced by a Gore-Tex prosthesis.

The 72-year-old patient was suffering from a liver metastasis located in segment I. The metastasis had infiltrated the caudal walls of the right, middle, and left hepatic vein (Figure 2). The tumor was only resectable together along with part of the inferior caval vein and parts of the liver veins; therefore an *ex situ* liver resection was planned. After laparotomy and exclusion of extrahepatic tumor manifestation, the veno-venous bypass was started after integration of two leukocyte adhesion filters in all four patients. During the liver mobilization phase, venous blood was filtrated continuously (in all four patients) after infradiaphragmal inferior vena cava (IVC) clamping. After total hepatectomy, a bench resection of the segments I, IV to VIII, and reconstruction of the middle and left hepatic vein and the IVC was done (Figure 3). During the *ex vivo* surgical procedure, the liver was perfused in 30 minute intervals through portal vein and hepatic artery using histidine-tryptophan-ketoglutarante solution. The auto-transplantations of the remnant liver followed the techniques applied in cadaver liver grafting.

#### *Case 2*

The 66-year-old patient was suffering from multiple liver metastasis located in segments IV, VII, and VIII with suspected infiltration of the left branch of the portal vein.

#### *Case 3*

The 49-year-old patient was suffering from recurrent liver metastasis located segment IVb with suspected infiltration of the portal vein. The patient had already gone through major liver surgery twice before.

#### *Case 4*

The 62-year-old patient was suffering from recurrent liver metastasis located in segment VI with suspected infiltration of the right branch of the portal vein. He had already gone through liver segmentectomy before.

### Blood sampling and immunohistochemistry

Blood samples for determination of WBC, platelet, free hemoglobin, and haptoglobin count were taken prior to operation and during the bypass circulation after 10, 20, 30, 40, 50, and 60 minutes, and on each postoperative day (POD).

Detection of cytokeratin positive (CK+) cells from venous blood (sampling prior surgery, during mobilization of the liver on the bypass and after filter passage, 3×9 mL aliquots) was achieved after Ficoll® (Pharmacia, Germany) density gradient centrifugation (669 g, 4°C, 30 minutes) by immunohistochemical labelling with an anti-cytokeratin antibody and microscopic analysis, regarding cell-morphologic parameters (Epimet-kit®, Micromet, Germany) [16-18].

## Results

An R0 resection was reached in all cases. The mean operation time was 8.2 hours; the mean bypass time was 1.2 hours for the *in situ* procedures, 6 hours in the *ex situ* case; mean filtration time was 39 minutes. The cardio-pulmonary situation remained stable during extracorporeal circulation in all cases.

No hemolytic reactions were noticed; parameters for hemolysis as free hemoglobin and haptoglobin remained within the normal range.

**Figure 4:**
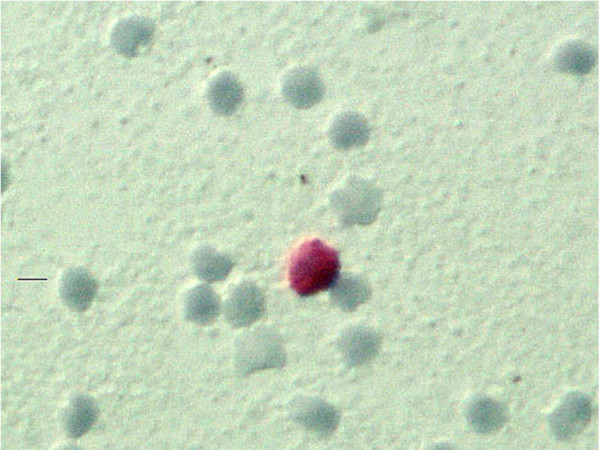
The image shows a cytokeratin positive (CK+) cell in hepatic venous blood (magnification 400×).

A significant perioperative reduction of the WBC count after starting filter passage was observed in all cases. After 30 minutes of filtration, the mean loss of 78% WBC was reached; the lowest value was 1.5 WBC per μL of blood in one case. The WBC increased to physiological values in all four cases within 48 hours after operation. CK+ cells – suspected as malignant cells – were detected in one case prior to operation, and in three cases during mobilization of the liver in venous blood before filter passage (Figure 4).

### Case 1

**Figure 5:**
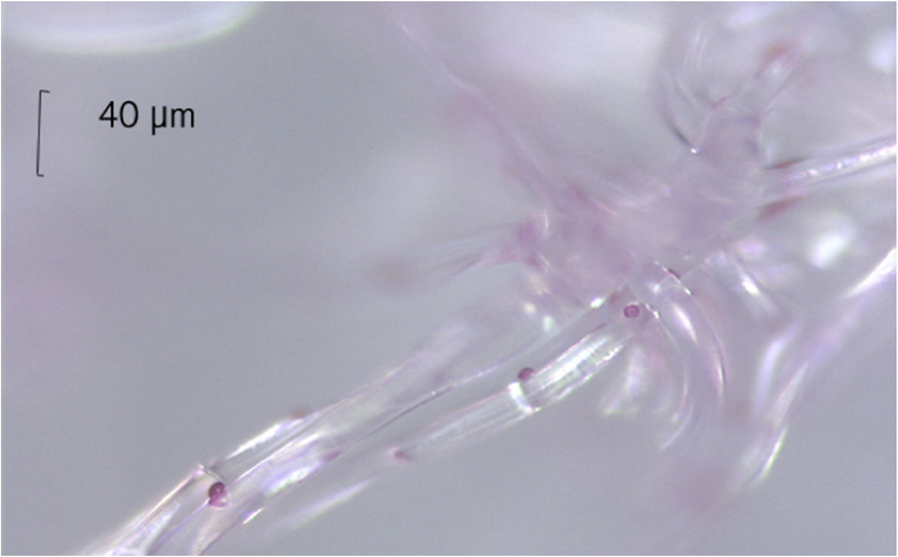
**Polarized light microscopy.** Microscopic analysis (20× magnification) of filter medium showing cytokeratin positive (CK+) cells.

The postoperative course was uneventful. Histology revealed multiple liver metastasis of an adenocarcinoma and a lymph node metastasis within the falciform ligament. CK+ cells, suspected as malignant cells, were detected in blood samples taken during mobilization of the liver and within the filter medium fibers which were analyzed microscopically (Figure 5). Comparable cells were not detected in blood samples from the filter outflow. The patient was discharged on the 27^th^ POD in a good general condition. The patient died 22 months after liver resection due to hepatic tumor recurrence.

### Case 2

The postoperative course was uneventful. Histology revealed two metastases of an adenocarcinoma within the liver (6 and 8 cm∅). CK+ cells were not detected in any blood sample. The patient was discharged on the 14^th^ POD. He died 21 months after the operation; 6 months prior to death pulmonary and cerebral metastases were detected, tumor recurrence within the liver was not observed.

### Case 3

The postoperative course was complicated by cardiopulmonary insufficiency. CK+ cells were detected in blood samples taken during mobilization of the liver and within the filter medium fibers. One suspect cell was also detected in blood samples from the filter outflow. The patient died on the 26^th^ POD. Post-mortem analyzes showed a myocardial infarction and endocarditis.

### Case 4

The postoperative course was uneventful. Histology revealed multiple liver metastases of an adenocarcinoma. CK+ were detected in blood samples taken during mobilization of the liver and within the filter medium fibers. Comparable cells were not detected in blood samples from the filter outflow. The patient was discharged on the 11^th^ POD. The patient is alive and in a good general condition without signs of tumor recurrence 26 months after operation.

A detailed summary of surgical procedures and intraoperative findings is shown in Table 2.

**Table 2:** Type of resection and perioperative findings

**Case**	**Resection**	**Vascular reconstruction**	**Technique**	**LG6**	**CK + A**	**CK + B**	**CK + C**	**BM**
**1**	I, IV-VIII	IVC, Liver veins by IVC patch and Gore-Tex prosthesis	*Ex situ*	47 min	-	+	-	nd
**2**	III, IV-VIII	LPV by venous patch	*In situ*	27 min	-	-	-	nd
**3**	IVb	RPV by venous patch	*In situ*	23 min	+	+	(+)	+
**4**	V-VIII	-	*In situ*	58 min	-	+	-	nd

Surgical procedures: resected liver segments, vascular reconstruction (*IVC* inferior vena cava, *LPV* left portal vein, *RPV* right portal vein), technical procedure (*ex* or *in situ* resection), duration of filtration with LG6 filter.

Perioperative findings: cytokeratin positive (CK+) cells within preoperative (A), intraoperative (B) and filter-outflow (C) blood samples; bone marrow (BM) status for CK+ cells. *Nd* no data.

## Discussion

Leukocyte depletion filters, used for preparation of leukocyte-poor red cell or platelet concentrates, have been evolving continuously [19]. Despite an evident capacity increase in leukocyte removal, most of the mechanisms of cell removal still remain unclear [20]. Multiple factors seem to be involved. The depletion of WBC is based on depth-filtration in a fiber-network with a possible subsequent adhesion to the fibers after activation [20]. There is evidence for a potential removal capacity also for malignant cells from whole blood [21]. The clinical use of leukocyte depletion filters has been established throughout different clinical disciplines. For years, leukocyte depletion filters have been routinely used in extracorporeal circulation systems, namely heart-lung machines for open heart surgery in order to reduce the ischemic reperfusion injury [12].

In contrast to the regulations for safety of blood products with a need of complete tumor cell depletion in transfusion medicine, a significant reduction of disseminating tumor cells during resection of tumor tissue in surgical oncology could indeed be helpful for the patient [22,23]. Although the characterization of tumor cells detected intraoperatively is still incomplete, there is evidence for lower survival rates in patients with disseminated tumor cells [24-26].

The cellular adhesion capacity of a filter medium is based on its saturation kinetics (Pall Company, manufacturer data). In the initial filtration period, a maximum of cells are removed from the circulating blood, exhausting filter capacity after approx. 20 minutes of filtration time. This effect also occurred in our study within the first 30 minutes of filtration time. In all cases, filtration within the veno-venous bypass up to 58 minutes (using two filter cartouches) was uneventful. Although a significant WBC depletion was achieved, critical leucopoenia complicated by postoperative infections was not observed. For safety reasons, the coagulation cascade should not be activated during filter medium passage. Gu et al. could not observe an activation of complement factors or increasing elastase or thromboxan concentrations in filter systems using polyester diaphragms [12]. In our own preclinical animal model [13] and in the presented cases, no signs of hemolysis were observed.

It remains more or less unclear which mechanisms of leukocyte depletion filters are involved in consecutive tumor cell depletion. Apart from the selection of the respective filter medium, the ability for tumor cell depletion also depends on the cell entity. Edelman et al. [23] proved a tumor cell-depleting effect using isolated tumor cells of urothelium carcinoma for the leukocyte filter system RC-400® (Pall, Germany) under *in vitro* conditions. Perseghin et al. [27] reported a similar clinical observation in patients with lung carcinoma, filtering cell saver blood from the surgical site using leukocyte depletion filters. Catling et al. showed, in 50 consecutive analyzed gyn-oncology patients, that no remaining viable tumor cell could be found after filtration of cell saver reservoir blood by leukocyte filters [28]. In our own preclinical work we could state under *in vitro* conditions that the leukocyte depletion filters used in this study reduced the number of native human tumor cells of colorectal carcinoma from blood-group-compatible full blood [11].

However, in our opinion, the liver mobilization phase carries the highest risk for tumor cell dissemination during surgery. During ‘conventional’ hepatic resection, complete mobilization of the right lobe of the liver, with the right hepatic vein controlled outside the liver before parenchymal dissection, has been advised by most surgeons. This helps to reduce the amount of surgical blood loss – unfortunately, it also includes manipulation of the tumor-bearing parts of the liver. The ‘non-conventional’ anterior liver resection technique is an attempt to avoid prolonged manipulation of the hepatic lobes, causing impairment of the afferent and efferent circulation. The technique involves initial completion of parenchymal dissection before the right lobe is mobilized. Belghiti et al. described a promising modification of this technique, using a tape introduced under the anterior surface of the IVC to ‘hang’ the liver and resulting in a safe approach to right hepatectomy without liver mobilization [29]. The anatomical basis of this ‘hanging maneuver’ has been thoroughly evaluated [30,31] and it has even been proposed to be safe in a modified approach for left hepatectomy by Suh et al. [32]. Previously, the advantage of the anterior over the conventional approach has been documented for the effects of tumor cell dissemination by Liu et al. in patients suffering from hepatocellular carcinoma [33]. Thus, according to the long established ‘no-touch’ philosophy, as little manipulation as possible seems a logical step also in oncologic liver surgery. In the presented study ‘non-conventional’ extended veno-venous bypass liver surgery was needed for oncological and safety reasons. Although one could argue that indications for *in*, *ex*, and *ante situ* techniques are rare, there is a small group of patients who could benefit from these techniques and would be otherwise non-resectable [34-36]. When a veno-venous bypass is used, leukocyte filters can be easily integrated.

## Conclusions

In summary, this study clearly showed that intraoperative filtration of potential malignant cells is technically feasible. Due to these results, further clinical studies are justified. This also may help correlate prognostic value and patient survival. Further differentiated information by closer characterization of entrapped and eventually passed tumor cells is needed, as well as the development of more differentiated surgical techniques using filtration technology. Combining filter systems with new surgical techniques requiring less manipulation and molecular target therapy against circulating tumor cells seems an especially promising approach to reduce tumor recurrence after liver resection.

### Consent

Written informed consent was obtained from the patient for the publication of this report and any accompanying images.

## Abbreviations

CK+: Cytokeratin positive; IVC: Inferior vena cava; POD: Postoperative day; WBC: White blood cells.

## Competing interests

The authors declare having no conflict of interests. We declare also to have had no stocks, shares or any financial relationship with any company mentioned in the article.

## Authors’ contributions

KO performed the surgical procedures and made substantial contribution to conception and design of the manuscript. GS made substantial contribution to analysis and interpretation of data and participated in drafting the article. MD made substantial contribution to analysis and interpretation of data and participated in revising the article critically for important intellectual content. PK carried out all laboratory studies including cytological analysis. NF made substantial contribution to conception and design of the manuscript and was actively involved in surgical procedures. All authors read and approved the final manuscript.
